# Community‐based care for people with chronic knee and hip pain: Preliminary clinical outcomes and healthcare utilisation for ESCAPE‐pain

**DOI:** 10.1002/msc.1847

**Published:** 2023-11-27

**Authors:** Michael Hurley, Francesca Thompson

**Affiliations:** ^1^ Insitutute for Population Health and Research St George's University of London and Kingston University London UK; ^2^ Orthopeadic Research UK London UK

**Keywords:** community‐based, *ESCAPE‐pain*, joint pain

## Abstract

**Background:**

Joint pain impairs physical and psychosocial wellbeing, quality of life (QoL) and has a significant socioeconomic impact. *Enabling Self‐management and Coping with Arthritic Pain using Exercise*, *ESCAPE‐pain*, is a rehabilitation programme that mitigates the wide impacts of joint pain. Financial, logistical and workforce constraints in health systems severely limit access to the programme. Delivering the programme by trained exercise professionals in community venues could increase access and reduce costs.

**Methods:**

Four hundred eighty‐two exercise professionals were trained to deliver *ESCAPE‐pain* at community sites to people >55 years with chronic knee or hip pain. Pain, physical function, QoL, self‐reported activity, mental wellbeing and healthcare utilisation (consultations, investigations, treatments, medication) were measured before, immediately after and 6 months after the programme.

**Results:**

One thousand four hundred ninety‐two people (mean age 70 years) were recruited. *ESCAPE‐pain* improved pain, function, QoL, mental wellbeing and objective physical function (*p* < 0.0001). Before the programme, only 24% of participants were classified as ‘fairly active/active’ (doing ≥30 min activity/week); after the programme, 78% were classified as ‘fairly active/active’; 6 months later, 69% were still ‘fairly active/active’. Participants used less healthcare after *ESCAPE‐pain*, resulting in savings of £326.16/participant.

**Conclusions:**

Older people with chronic joint pain were willing to attend *ESCAPE‐pain* when delivered by exercise professionals in community centres, and it was found to be as effective as when delivered by physiotherapists in hospitals. Delivering *ESCAPE‐pain* in the community could facilitate access to effective care and on‐going support to sustain the benefits of healthcare programmes, producing a more efficient use of health and community resources.

## 1 BACKGROUND

Worldwide osteoarthritis (OA) is one of the leading causes of joint pain, impaired mobility, physical function, psychosocial wellbeing and quality of life (QoL) (Hunter & Bierma‐Zeinstra, 2019; Vos et al., 2020). It also has a significant socioeconomic impact (Leifer et al., 2022), for example, in the UK OA accounts for over £3billion in lost productivity (Chen et al., 2012). These personal, societal and economic costs are increasing rapidly as more people live longer, but are less active and often overweight (Hunter & Bierma‐Zeinstra, 2019; Public Health England, 2018; Versus Arthritis, 2019). Consequently, joint pain due to OA is a major and rapidly growing public health problem (Kiadaliri & Englund, 2021; Public Health England, 2018; Versus Arthritis, 2014; Vos et al., 2020) that was exacerbated by the COVID19 pandemic (Oussedik et al., 2021).

All international management guidelines (National Institute for Health and Clinical Excellence, 2022; Rausch Osthoff et al., 2018) recommend that people with joint pain take part in regular physical activity, as physical activity reduces joint pain and mitigates its physical and psychosocial impacts (Hurley et al., 2018; Krause et al., 2019). *Enabling Self‐management and Coping with Arthritic Pain using Exercise*, *ESCAPE‐pain*, is a rehabilitation programme that integrates information and advice with a graduated exercise programme, that reduces pain and improves mobility, physical and mental health wellbeing, and increases people's confidence in their ability to self‐manage joint pain (Hurley et al., 2007, 2010). Optimising non‐operative treatment might help reduce waiting times for outpatient treatment and surgery.

Until recently *ESCAPE‐pain* was delivered by physiotherapists in hospital outpatient departments. Unfortunately, financial, logistical and workforce constraints severely limit the accessibility and delivery of the programme through health systems, severely restricting the number of people who can benefit from the programme. Moreover, health systems have very limited capacity to support people to maintain their physical activity and hence the benefits it has helped them attain after they have completed the programme. To improve access to the programme and provide opportunities for on‐going support to sustain the benefits of the programme, we trained exercise professionals to deliver *ESCAPE‐pain* in community venues (leisure centres, community halls, etc).

This study evaluated whether *ESCAPE‐pain* replicated improved clinical outcomes when delivered in community venues and reduced healthcare utilisation.

## 2 METHODS

### 2.1 Programme facilitators

As part of Sport England's ‘*Active Ageing*’ initiative *ESCAPE‐pain* was adopted as a programme that could increase physical activity in older people. 482 exercise professionals from 17 community leisure organisations were trained to deliver over 200 *ESCAPE‐pain* programmes at 75 sites across England. To deliver the programme, exercise professionals needed to have Level 3 Exercise Referral qualifications and 150 h of experience supervising people with health conditions (such as exercise on referral, cardiac or pulmonary rehabilitation programmes). They had to attend a 1‐day training course that described the ethos, format and content of *ESCAPE‐pain*, and ensured they delivered a standardised programme.

### 2.2 Programme participants

People recruited onto the programme had to be 55 years or older, have knee and/or hip pain for at least 6 months, and no serious unstable physical or mental health problem that prevented their participation in an exercise programme.

### 2.3 Intervention

*ESCAPE‐pain* is a rehabilitation programme for people with knee and/or hip pain that integrates information, advice and support, with a progressive, challenging exercise regimen. The programme helps participants understand their problem, dispels erroneous health beliefs, advises them what (not) to do, and enables them to experience the benefits of exercise and take control of their symptoms. Detailed descriptions of the programme are available (Hurley et al., 2007) www.ESCAPE‐pain.org), but briefly *ESCAPE‐pain* is delivered to groups of 8–12 people aged 45 years and older who attend 12 sessions (twice a week for 6 weeks) led by a trained facilitator. Each session comprises:

- a ∼25‐min **education** component that takes the form of a themed discussion (covering causes of joint pain, prognosis, advice, and pain self‐management/coping strategies, such as heat/ice, rest‐activity cycling, relaxation) with behavioural change techniques (goal‐setting, action/coping planning, positive feedback, etc) threaded into the programme, and emphasises that exercise is a safe, effective way to reduce pain and increase function;
- a ∼40‐min supervised **exercise** component where participants undertake a personalised, progressive exercise regimen to increase strength, endurance and function.

The blend of information‐giving, support, shared‐learning and experiential learning alters people's beliefs about joint pain and its impact and encourages them to change their behaviour and adopt healthier lifestyles, especially by increasing their participation in regular physical activity.

### 2.4 Outcomes

*Pain*, *function and QoL* related to knee or hip pain was assessed using the short version of the Knee or Hip Osteoarthritis Outcome Score (K/HOOS) (Braaksma et al., 2020; Davis et al., 2009). This widely used, reliable and responsive outcome asked standardized questions about pain, function and QoL. Answers were assigned a score from 0 (no problems) to 4 (extreme problems) and a normalised score (100 = no symptoms/problems, 0 = extreme symptoms/problems) was calculated for each subscale. K/HOOS was administered before starting (baseline) and immediately after completing the programme.

*Self‐reported activity levels* (minutes/week) were measured using the Short Active Lives (SAL) Questionnaire, which asked participants about the amount of time they spent doing various activities in the past week (Sport England, 2023a). Participants reporting less than 30 min activity sufficient to increase their breathing rate were classified as ‘inactive’, those completing 30–149 min of activity were classified as ‘fairly active’ and those completing 150+ minutes classified as ‘active’. The SAL was completed at baseline, immediately after completing the programme and 6 months later.

*Objective physical function* was assessed using the “30 s sit‐to‐stand” test, that counted the number of times a participant, with their arms folded, could stand up and sit down from an armless chair in 30 s (Alcazar et al., 2018). The ‘sit‐to‐stand’ test was administered at baseline after completing the programme and 6 months later.

*Mental wellbeing* was measured using the Short Warwick Edinburgh Mental Wellbeing Scale (SWEMWBS) (Bass et al., 2016) before and after each participant completed the programme.

### 2.5 Data Analysis

Data are presented as means with 95% confidence intervals (95% CI). Pre‐programme versus post‐programme differences were compared using paired *t*‐tests (Stata Corp v16). Statistical significance was set at *p* < 0.5.

*Healthcare utilisation* by programme participants was gathered during a telephone interview using the Client Services Receipt Inventory (Personal Services Social Research Unit, 2023). Participants were asked about the number of consultations with health professionals, medical interventions and medication related to their knee or hip pain they had used in the 6 months before beginning *ESCAPE‐pain* (baseline assessment, *n* = 204) and 6‐months (*n* = 137) after completing the programme. After eliminating participants who had missing data, analyses were conducted on data from 108 participants for whom we had complete record healthcare utilisation for the 6 months prior to baseline assessment *and* 6 months immediately after completing *ESCAPE‐pain*. To calculate healthcare costs in the 6 months before and after the programme, NHS unit costs taken from the Unit Costs of Health and Social Care 2022 Manual complied by the Personal Services Social Research Unit (Jones et al., 2022) and NHS England cost databases (NHS England, 2022) total costs were calculated by multiplying the unit costs by the number of units used.

## 3 RESULTS

1492 people were recruited (mean age 70 years; 72% female; 80% considered knee pain their predominant problem) of whom 1364 (91%) attended at least 8 sessions of an *ESCAPE‐pain* programme.

### 3.1 Pain, function and QoL

Prior to the programme levels of pain, function and QoL were similar regardless of whether participants considered their knee or hip joint to be most painful. After the programme knee and hip related pain, function and QoL improved by approximately 10 points (*p* < 0.0001; Table 1) again regardless of whether the knee or hip joint was predominantly affected. Because of the similarity between the baseline knee and hip joint pain and size of the improvements in pain function and QoL following the programme, outcome data for knees and hips were combined (Table 1).

**TABLE 1:** Clinical outcomes before, after and 6 months after participation in the ESCAPE‐pain programme.

Variable	Pre‐programme			Post‐programme			6‐month follow‐up		
	*n*	Mean	95% CI	*n*	Mean	95% CI	*n*	Mean	95% CI
K/HOOS ‐ function	1020	51.43	50.28 to 52.58	1020	61.26*	60.10 to 62.43			
K/HOOS Pain	1034	47.57	46.52 to 48.63	1034	57.88*	56.78 to 58.98			
K/HOOS QoL	1030	34.88	33.76 to 36.00	1030	44.75*	43.56 to 45.94			
SWEMWBS	997	25.3	24.96 to 25.63	997	27.24*	26.95 to 27.54			
Sit‐to‐Stand	1360	8.18	7.97 to 8.38	1106	12.18*	11.89 to 12.46	195	12.14*	11.32 to 12.96
SAL (mins)	1391	74.36	61.3 to 84.4	1135	305.31*	268.95 to 341.68	489	192.76*	163.89 to 221.64

Abbreviations: 95% CI, 95% confidence interval; K/HOOS, Knee/Hip Osteoarthritis Outcome Score; *n*, number of participants included; P, probability level; QoL, quality of life; SAL, short active lives; SWEMWBS, Short form Warwick and Edinburgh Mental Wellbeing Scale.

*p < 0.0001.

### 3.2 Mental wellbeing and objective physical function

After the programme participant's mental wellbeing improved by 2 points (pre‐programme 25.3 points 95% CI 25–25.6 vs. 27.2 points 95% CI 27–27.5 post‐programme; *p* > 0.0001; Table 1). The number of sit‐to‐stands performed in 30 s increased from 8.2 (95%CI 8–8.4) to 12.2 (95% CI 11.9–12.5) and was sustained 6 months after the programme (12.1 95% CI 11.3–13; *p* < 0.0001; Table 1).

### 3.3 Physical activity levels

Before the programme, only 24% of participants were classified as fairly active/active (doing ≥30 min activity/week), and participants reported taking part in an activity that raised their breathing rate for about 74.4 min (95% CI 61.3–84.4) minutes/week (Table 1). After completing the programme, the average time participants took part in activities that raised their breathing rate rose to 305 min/week (95% CI 268.9–341.7; *p* < 0.0001; Table 1), and 78% of participants were classified as fairly active/active. Six months later, participants were reporting being active for 192.8 min/week (95% CI 163.9–221.6; *p* < 0.0001; Table 1) and 69% of participants were classified as fairly active/active.

### 3.4 Healthcare utilisation

As stated above, healthcare utilisation in the 6 months before and after the programme was calculated for each participant and from the expenditure and savings following participation on the programme estimated (Table 2). Of the 108 for whom there were baseline and 6‐month data were available, 77% were female, mean age 71 years. The general pattern was that programme participants accessed and used less healthcare (consultations, investigations, treatments, medication) in the 6 months following the programme, compared to their usage in the 6 months prior to the programme (Table 2). For example, participants with data for 6 months preceding and 6 months after completing *ESCAPE‐pain* 64 participants attended 115 GP consultations for knee/hip pain preceding the programme, whereas 38 participants attended 57 GP consultations after the programme, a reduction of 58 consultations. On average, each participant had 1 GP consultation in the 6 months prior to *ESCAPE‐pain*, compared with 0.5 GP consultation per participant after the programme (Table 2).

**TABLE 2:** Healthcare utilisation, unit costs and healthcare savings in the 6 months before and after participation in the ESCAPE‐pain programme.

Variable	Unit cost	6 months prior to baseline (*n*‐108)		Baseline to 6‐month (*n*‐108)		Baseline to 6 months (*n* = 108)		
			Total unit costs		Total unit costs	Number of units saved	Comparative cost saving	
Number of participants consulting GP		64 (59%)		38 (35%)				
Total number of GP consultations^a^	£ 41.00	115	£ 4715.00	57	£ 2337.00	58	£ 2378.00	
GP consultations per respondent		1.07		0.53				
Outpatient consultation^b^	£ 272.60	22	£ 5997.20	14	£ 3816.40	8	£ 2180.80	
Physiotherapy^c^	£ 73.14	41	£ 2998.74	14	£ 1023.96	27	£ 1974.78	
Investigations/Treatments		61		23				
Surgery^d^	£ 7205.00	5	£ 36,025.00	2	£ 14,410.00	3	£ 21,615.00	
X‐ray^e^	£ 38.28	38	£ 1454.64	14	£ 535.92	24	£ 918.72	
MRI^f^	£ 156.05	9	£ 1404.45	4	£ 624.20	5	£ 780.25	
Injection^g^	£ 233.80	9	£ 2104.20	3	£ 701.40	6	£ 1402.80	
No. participamts prescribed medication		64		48				
No of prescribed medications^h^	£ 33.12	300	£ 9936.00	180	£ 5961.60	120	£ 3974.40	
Total costs			£ 64,635.23		£ 29,410.48		£ 35,224.75	Total savings
Costs per participant			£ 598.47		£ 272.32		£ 326.16	Saving per participant
Total costs extrapolated to 1364 participants			£ 816,319.02		£ 371,443.47		£ 444,875.55	Savings 1364 participants

*Note*: Taken from worksheets from database of National Schedule of NHS Costs Year 2021/22 (https://www.england.nhs.uk/costing‐in‐the‐nhs/national‐cost‐collection) unless stated.

Abbreviations: GP, General Practitioner; MRI, Magnetic Resonance Image; *n*, number.

^a^ GP consultation £41 per 9.22 min consultation (taken from “Unit Costs of Health and Social Care 2022”, PSSRU; Table 9.4.2; Pg 70).

^b^ Hospital Outpatient Consultation (Outpatient; Consultant led; Rheumatology Services; Non‐admitted face‐to‐face attendance, First).

^c^ Community Health Services (Allied Health Professionals; Physiotherapist, Adult, One‐to‐One).

^d^ Surgery—Total Health Resource Group (Very Major Knee Procedures for Non‐Trauma with CC Score 0–1).

^e^ X‐ray—Directly Accessed Diagnostic Services (Direct Access Plain Film).

^f^ MRI—Diagnostic Imaging (Magnetic Resonance Imaging Scan of One Area, without Contrast, 19 years and over).

^g^ Injection—Outpatient procedures (Injection of Therapeutic Substance into Joint for Pain Management).

^h^ Prescription costs per consultation—(taken from “Unit Costs of Health and Social Care 2022”, PSSRU; Table 9.4.2; Pg 70).

From these data, the cost savings to the NHS were estimated to be £326.16/participant over the first 6 months after *ESCAPE‐pain* (Table 2). Extrapolating this to the estimated 1364 participants an indicative cost saving to the NHS of £444,875.55.

## 4 DISCUSSION

In this study, older people with chronic knee and/or hip pain were willing to attend a rehabilitation programme, *ESCAPE‐pain*, delivered by exercise professionals in community leisure centres. The programme was safe, popular, effective and reduced healthcare utilisation.

To be useful and widely adopted, healthcare programmes must be safe, needed, effective and affordable. The fact that people were willing to attend *ESCAPE‐pain*, it was well received and there were no adverse events shows it was safe and wanted. The improvements reported in pain, physical function, activity levels, mental and emotional wellbeing and QoL were similar to those found when *ESCAPE‐pain* is delivered by healthcare professionals in clinical settings (Hurley et al., 2007; Jessep et al., 2009).

The two outcomes we were able to measure 6 months after completing the programme (physical function, activity level) suggest these benefits can be sustained (Hurley et al., 2012).Of special note is that whereas only a small minority (24%) of participants could be categorised as being ‘active’ before *ESCAPE‐pain* (active for 30 min/week), 78% could be categorised as being active after the programme and most (69%) remained active over the subsequent 6 months. Given that regular physical activity is accepted to be an important self‐management strategy that enables people to control joint pain and its impacts (National Institute for Health and Clinical Excellence, 2022; Rausch Osthoff et al., 2018), sustaining behavioural change (i.e. regular participation in physical activity) is vital to maintain the benefits of healthcare programmes.

We attributed these improvements to enhanced ‘exercise self‐efficacy’ (Bandura, 1977; McAuley et al., 1993; Shields & Brawley, 2006)—a person's improved understanding of the role exercise plays in mitigating pain and its impact, which enables them to be more active, thereby controlling their problems. After the programme participants describe being able to walk better, farther and for longer, resuming previous activities and feeling more optimistic about the future (Hurley et al., 2010). Increased self‐efficacy in their ability to use exercise to lessen the impact of joint pain may reduce people's reliance on healthcare professionals; consequently, they require fewer consultations, investigations, medication and interventions (Hurley et al., 2007). This is reflected in reduced healthcare costs (£326/person) in the 6 months after the programme. Extrapolated to the 1364 participants in the service evaluation *ESCAPE‐pain* would reduce healthcare costs by nearly £445,000. If extrapolated to the millions of people with joint pain, it could produce huge savings to healthcare budgets as well as people's personal significant out‐of‐pocket expenses (Kotlarz et al., 2009).

Reaching the huge number of people with joint pain cannot be achieved with the limited resources of healthcare systems. Community providers have greater capacity (facilities, workforce) to meet the demand for better management of joint pain. They also have the ability to provide ongoing support and opportunities for people to participate in regular physical activity after they have completed the programme thereby retaining its benefits. Furthermore, the community leisure sector has the ambition to develop collaborative partnerships with local healthcare systems in order to increase their presence in the health sector (Sport England, 2023b; UKActive, 2023). This is partly to have greater relevance to their local communities, but also to create revenue streams and grow their businesses. Consequently, the community organisations in this study have devised innovative, “low‐cost/no‐cost” ways of delivering ESCAPE‐pain and post‐programme classes that enable participants to maintain its benefits and enable long‐term financial sustainability of the programme (Hurley et al., 2021). Similarly, the NHS ‘Long Term Plan’ is to manage more people in their local communities with community‐based interventions, and has established Integrated Care Boards that link local health and community sectors (NHS England, 2019). As a result of these policies and initiatives, two of the community providers in our study established formal partnerships with local healthcare commissioners to deliver *ESCAPE‐pain* to address the unmet demand for more convenient access to better care, relieve the burden on struggling healthcare systems, and generate opportunities for community providers to expand their involvement in healthcare.

### 4.1 Strengths and limitations of the study

This study is a service evaluation of a well‐established programme delivered under ‘real‐world’ conditions. Its main strength is its ‘real‐world’ nature: we recruited a heterogeneous, but representative population from large commercial organisations and small local social enterprises in inner city and rural settings, with varied cultural and socioeconomic profiles from across the UK. This heterogeneity increases the probability that our findings will be replicated when the programme is applied in other centres.

A limitation of the study is the lack of design factors (control group, randomisation, missing data, etc) which increase the likelihood of introducing biases that could affect the results. For example, recruiting people to initiatives that interest them and require investment of their time and effort may result in ‘volunteer bias’ which can inflate the outcomes. Whether and how much volunteer bias may have affected the results of this study cannot be known. But the large number of people in the study and the size of the improvements may have limited volunteer bias.

In addition, the lack of evidence on the economic consequences of exercise in MSK conditions, testifies to the difficulty of conducting studies in this area. Resource limitations meant we could only gather and longitudinally follow economic data from a relatively small number of people. So although there is an indication of potential savings, reflecting what we and others have found previously, these should be seen as rudimentary data that could be used to support further work.

Our clinical and economic findings need to be corroborated in larger, robustly designed trials, realist evaluations and/or from routine collection of “real world” data.

In summary, older people with knee and/or hip pain who may not be, and/or worried about, participating in regular physical activity, are willing to attend a healthcare programme, *ESCAPE‐pain*, delivered by exercise professionals in community settings. The programme was safe and effective and can reduce healthcare utilisation and produce sustained behavioural changes in physical activity levels that could maintain *ESCAPE‐pain's* short‐term benefits.

Improving access to community‐based programmes could have benefits for all concerned. For health care providers, it relieves pressure on their facilitates, workforce and finances. For community organisations, it can help them benefit their local community by increasing their relevancy, facilitate partnership working with local health systems and generate revenue streams. Most importantly, for the millions of people suffering from joint pain, it increases access to effective care and on‐going support to provide sustained relief from chronic joint pain and its impact.

## AUTHOR CONTRIBUTIONS

Michael Hurley was involved in developing the concept of the study, obtaining funding, overseeing its conduct, data collection, collation and analysis and was the main author of the report. Francesca Thompson was involved in the development of the concept of the study, data collation and extensively contributed to drafts of the manuscripts.

## CONFLICT OF INTEREST STATEMENT

The authors report no conflicts of interest.

## ETHICS STATEMENT

As this was an evaluation of an existing programme, ethical approval was deemed not to be required. The reason we were conducting the study was explained to all interviewees, and it was emphasised that they had the right to refuse to be interviewed, withdraw from an interview and/or ask for their interview to be deleted at any time.

## Data Availability

The data supporting the findings of this study are available from the corresponding author, [Hurley], upon reasonable request.
